# The Hypodermic Injection of Morphia

**Published:** 1880-02

**Authors:** 


THE HYPODERMIC INJECTION OF MORPHIA. Its History, Advantages and Dangers. [Based on the experience of 360 physicians.] By H. H. Kane, M.D. New York: CHAS. L. BIRMINGHAM & Co. 1880.

The author, while acknowledging the benefits of this popular manner of relieving suffering, insists on the certainty of both immediate and remote disadvantages. We are of that class who believe that, intrinsically, there is not the least danger in hypodermic medication. Accidents will happen, and that, too, despite any system or rules that may be instituted for the purpose of averting their casualties. We have heard many attempt to disparage the benefits and exaggerate the dangers of this plan of administering remedies, "one of whom" Dr. H. V. M. Miller, of this city, "is which;" but we have yet to hear sound, convincing arguments against it. Exceptions should not abrogate general rules, and details, individually, should not supersede general facts. The work before us is worthy perusal, and furnishes much data whereby to extend the dispute. The following questions are propounded by the author, with the hope that by a general reply certain unsettled questions may be cleared up:

1. In how many cases of delirium tremens, in what doses and with what result, have you used morphia hypodermically?

2. Have you used the drug in this manner in acute inflammatory affections of the respiratory organs, and with what result?

3. Have you used it in acute or chronic venal disease, and with what result?

4. Do you know of any deaths due to the subcutaneous injection of morphia? If any autopsy, what the result?

5. Have you had any serious cases of narcotism from the use of morphia in this manner?

6. Have you had any cases where the drug was thrown directly in the blood? What were the symptoms and what the treatment?

7. In what diseases have you used this method of administering morphia, and with what results?

